# Multiple structure alignment and consensus identification for proteins

**DOI:** 10.1186/1471-2105-11-71

**Published:** 2010-02-02

**Authors:** Ivaylo Ilinkin, Jieping Ye, Ravi Janardan

**Affiliations:** 1 Department of Computer Science, Gettysburg College, Gettysburg, PA, USA; 2 Department of Computer Science and Engineering, Arizona State University, Tempe, AZ, USA; 3 Department of Computer Science and Engineering, University of Minnesota, Minneapolis, MN, USA

## Abstract

**Background:**

An algorithm is presented to compute a multiple structure alignment for a set of proteins and to generate a consensus (pseudo) protein which captures common substructures present in the given proteins. The algorithm represents each protein as a sequence of triples of coordinates of the alpha-carbon atoms along the backbone. It then computes iteratively a sequence of transformation matrices (i.e., translations and rotations) to align the proteins in space and generate the consensus. The algorithm is a heuristic in that it computes an approximation to the optimal alignment that minimizes the sum of the pairwise distances between the consensus and the transformed proteins.

**Results:**

Experimental results show that the algorithm converges quite rapidly and generates consensus structures that are visually similar to the input proteins. A comparison with other coordinate-based alignment algorithms (MAMMOTH and MATT) shows that the proposed algorithm is competitive in terms of speed and the sizes of the conserved regions discovered in an extensive benchmark dataset derived from the HOMSTRAD and SABmark databases.

The algorithm has been implemented in C++ and can be downloaded from the project's web page. Alternatively, the algorithm can be used via a web server which makes it possible to align protein structures by uploading files from local disk or by downloading protein data from the RCSB Protein Data Bank.

**Conclusions:**

An algorithm is presented to compute a multiple structure alignment for a set of proteins, together with their consensus structure. Experimental results show its effectiveness in terms of the quality of the alignment and computational cost.

## Background

This paper presents an algorithm to compute a multiple structure alignment for a set of proteins and to generate a consensus structure. The algorithm is called MAPSCI, which stands for **M**ultiple **A**lignment of **P**rotein **S**tructures and **C**onsensus **I**dentification. MAPSCI addresses the problem of global structure alignment, which has also been considered by CE-MC [1], MAMMOTH [2], and MATT [3]. Specifically, MAPSCI computes an approximation to the multiple structure alignment that minimizes the so-called *Sum-of-Consensus distance (SC-distance)*, i.e. the sum of the pairwise distances between the consensus structure and each protein in the set (see the **Methods **section for the precise definition of *SC-distance*). Our experiments show that MAPSCI converges quite rapidly and produces alignments that compare favorably with the alignments produced by MAMMOTH and MATT. The consensus structures generated by MAPSCI are visually quite similar to the input proteins. Although the consensus structures are not real proteins, they could be used, for instance, as templates to perform fast searches through protein structure databases, such as the Protein Data Dank [4], to identify structurally similar proteins.

MAPSCI has similar structure to the algorithm of Ye and Janardan [5]. However, MAPSCI works directly on the coordinates of the *C*_*α *_atoms and produces true alignments; by contrast, the algorithm in [5] requires that the backbone vectors be translated to the origin, hence information about the relative positions of the *C*_*α *_atoms in ℝ^3 ^is lost and as a result the algorithm does not generate true alignments. The **Methods **section presents the mathematical and algorithmic framework of MAPSCI and provides the complete details where the two algorithms differ significantly; when there is an overlap the reader is referred to publication [5].

## Implementation

MAPSCI represents the input proteins and the consensus as sequences of triples of coordinates of the alpha-carbon (or *C*_*α*_) atoms along the backbone. It then computes a correspondence between the coordinate triples of the *C*_*α *_atoms in the different protein structures by choosing one of the proteins as the initial consensus and applying an algorithm that is analogous to the center-star method for multiple sequence alignment [6]. Next, MAPSCI derives a set of translation and rotation matrices that are optimal for the computed correspondence and uses these to align the structures in space via rigid motions and obtain the new consensus. The process is repeated until the change in *SC-distance *is less than a prescribed threshold. This iterative process is well-defined as it is shown in the **Methods **section that the *SC-distance *is non-increasing from one iteration to the next. The computation of the optimal translations and rotations and the new consensus is itself an iterative process that both uses the current consensus and generates simultaneously a new one.

Table 1 summarizes the algorithm in pseudocode form. The various steps in the pseudocode are described in more detail in the **Methods **section. The algorithm has been implemented in C++ and can be used stand-alone or run remotely via a web-based interface. The source code of the implementation is available for download from the project's website (see the **Availability **section). The implementation is organized as a library of algorithms and simple data structures that can be integrated in other projects. Examples of using the library within a C++ program are given in the README file of the source code distribution. The iterative process described above employs pairwise structure alignment as an intermediate step and the parameters that control the execution of the multiple alignment algorithm are the parameters for the underlying pairwise alignment algorithm. The current implementation uses the pairwise alignment algorithm described in [7]; however, other algorithms for pairwise structure alignment can be used instead.

**Table 1:** Algorithm MAPSCI: Multiple Alignment of Protein Structures and Consensus Identification

1. Choose initial consensus structure from . *i *← 0. *SC*^0 ^← ∞.
2. Do
3. if *i *= 0 then compute pairwise structure alignment between and every *P*_*j*_.
4. else use standard dynamic programming to align with every *P*_*j*_.
5. *i *← *i *+ 1.
6. Compute correspondence from the above alignments (either pairwise or dynamic programming) using center-star-like method.
7. Compute optimal translation matrix and optimal rotation matrix iteratively (Theorems 2 and 3). Transform *P*_*j *_by and for every *j *to obtain multiple structure alignment ℳ^*i*^. *SC*^*i *^← *SC*(ℳ^*i*^).
8. Post-process ℳ^*i *^by removing all columns consisting of only gaps.
9. Compute new consensus structure from ℳ^*i *^by Theorem 1.
10. Until .//*η *is a user-specified threshold (currently set at 0.0001)

## Results

### Web Server

**Figure 1:**
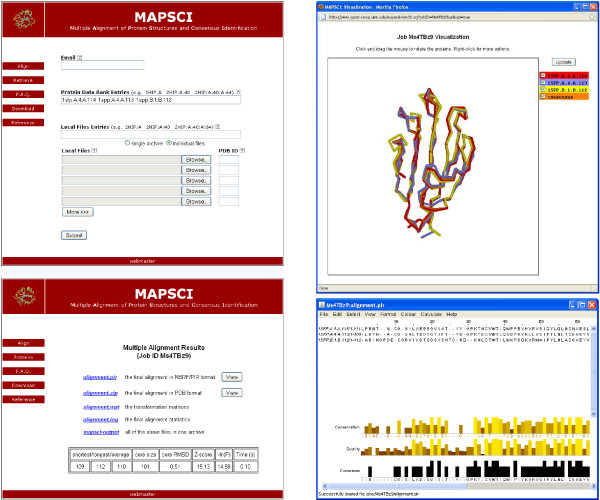
**Web server screenshots**. Screenshots from the web server: main page (top left), results page (bottom left), structure view (top right), sequence view (bottom right).

MAPSCI has been incorporated into a web server for remote access over the Internet (see Figure 1). This tool allows for protein structures to be uploaded from files on the local disk or retrieved from the Protein Data Bank (PDB) [4] by specifying their PDB ids. The results from the alignment are annotated in the standard NBRF/PIR format, which can be previewed online via the Jalview applet [8]. Integrated with the server is the molecular viewer applet Chemis 3D [9], which allows for visualization of the aligned protein structures.

The web server offers a simple interface that allows for remote access from within other software. Table 2 gives an example of using the programming language Python to retrieve the transformed coordinates (in PDB format) for the multiple alignment of the structures from the HOMSTRAD CUB family. Additional examples and the complete set of options for remote access can be found at the server web page (see the **Availability **section).

**Table 2:** Remote access to the server

import urllib2
url = "http://www.geom-comp.umn.edu/mapsci/align.cgi?wsget=pdb&rcsb=1sfp+1spp:A+1spp:B"
server = urllib2.urlopen(url)
output = file("alignment.zip", 'wb')
output.write(server.read())
output.close()
server.close()

An example of using the programming language Python to retrieve the transformed coordinates (in PDB format) for the multiple alignment of the structures from the HOMSTRAD CUB family. Additional examples and the complete set of options for remote access can be found at the server web page (see the **Availability **section).

### Comparison

As discussed earlier, there are many algorithms for multiple structure alignment. In general, it is difficult to make comparisons among them, since they operate under different sets of assumptions and problem formulations. We compare MAPSCI to two recent algorithms -- MAMMOTH [2] and MATT [3] -- which also work with coordinate triples, but employ a different objective function. Our experiments show that MAPSCI is competitive in terms of the sizes of the so-called conserved regions and runs significantly faster than the other two algorithms, hence can potentially scale to much larger datasets.

**Figure 2:**
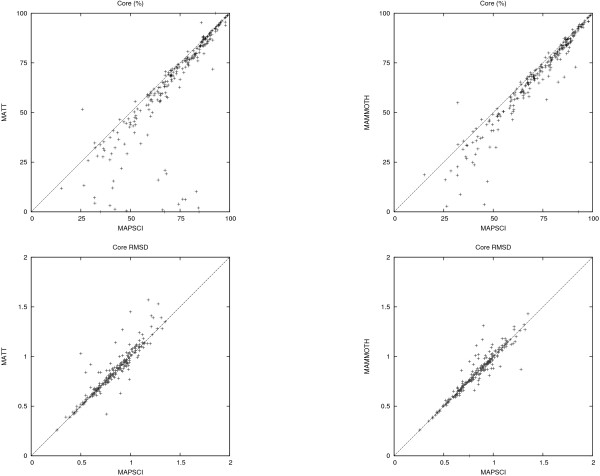
**HOMSTRAD dataset comparison**. Comparison based on the *strict core *metric (expressed in percent of the size of the shortest protein) and the *strict core RMSD *on the HOMSTRAD dataset.

**Figure 3:**
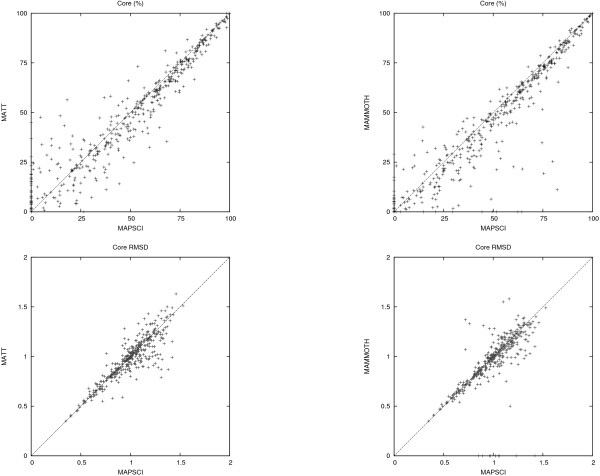
**SABmark dataset comparison**. Comparison based on the *strict core *metric (expressed in percent of the size of the shortest protein) and the *strict core RMSD *on the SABmark dataset.

The comparison is based on two benchmark datasets. The first dataset is compiled from the HOMSTRAD database [10], which is a curated database of structure-based alignments for homologous protein families and is considered the "gold" standard. The benchmark dataset consists of the 232 HOMSTRAD families that have at least 4 structures. The second dataset consists of the *superfamily set *in the SABmark database [11] (version 1.65). It contains 425 families with low to intermediate sequence similarity. The metrics considered in the comparison are the *strict core *(or just *core*) and the core RMSD. This follows the experimental setup in [2] where *strict core *is defined as "the set of positions with 100% conservation, and within 4.0 Å of each other in the final structural alignment in 3D". A similar metric is discussed in [12] and [13]. The results are summarized in Figures 2 and 3, which show the pairwise comparisons (MAPSCI, MAMMOTH), (MAPSCI, MATT) in terms of the core size (expressed in percent of the length of the shortest protein) and the core RMSD. Table 3 provides a comparison of the average core size and average core RMSD for the three methods on the benchmark datasets.

**Table 3:** Benchmark datasets performance

	HOMSTRAD		SABmark	
	Average Core (%)	Average Core RMSD	Average Core (%)	Average Core RMSD
MAPSCI	70.99	0.83_(*n *= 232)_	48.89	1.00_(*n *= 385)_
MAMMOTH	66.74	0.83_(*n *= 231)_	44.55	0.99_(*n *= 394)_
MATT	63.79	0.85_(*n *= 229)_	47.88	0.99_(*n *= 420)_

Statistics for the performance of the three methods on the benchmark datasets. The subscripts in the *Average Core RMSD *columns indicate how many values were used in computing the statistics, since the algorithms failed to compute a core for some of the data sets. For the *Average Core (%) *columns all reported values were used and therefore *n *= 232 and *n *= 425 for the HOMSTRAD and SABmark datasets, respectively.

In general, it is difficult to compare two algorithms based on these two metrics (larger cores tend to have larger RMSD). However, on the HOMSTRAD dataset MAPSCI outperformed MAMMOTH in 45% of the test cases and MATT in 59% of the test cases by computing alignments with both larger cores and smaller core RMSD. (MAMMOTH and MATT were better than MAPSCI on both metrics combined in 6% and 5% of the test cases, respectively). MAPSCI computed cores for all 232 test cases, while MAMMOTH failed to compute a core for one family (*bowman*), and MATT failed to compute a core for three families (*asp*, *lipocalin*, and *tln*).

On the SABmark dataset MAPSCI computed larger cores with better RMSD in 39% of the test cases when compared with MAMMOTH and in 37% of the test cases against against MATT. (MAMMOTH and MATT were better than MAPSCI on the two metrics combined in 15% and 26% of the test cases, respectively.) MATT was the most robust of the three algorithms and failed to compute a core in only five test cases; MAPSCI failed on 40 families and MAMMOTH failed on 31 families.

**Figure 4:**
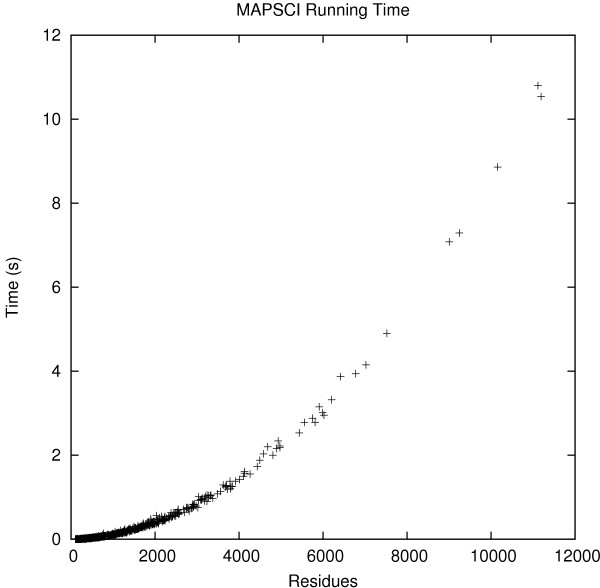
**Execution time**. The actual execution time of MAPSCI for all families in the benchmark datasets plotted in terms of the total number of residues per family.

MAPSCI took only 151 seconds to align the 425 families in the SABmark dataset and 85 seconds to align the families in the HOMSTRAD dataset. MAMMOTH took 1100 seconds on the SABmark dataset and 649 seconds on the HOMSTRAD dataset. By contrast, MATT took several hours to process the two datasets. Figure 4 shows the actual time taken by MAPSCI for all families in the benchmark dataset in terms of the total number of residues per family. The algorithm converges very quickly and can potentially scale to large datasets. The machine used for all experiments reported in the paper runs Ubuntu Linux 8.04 and has 4 GB of RAM with Intel^®^Core™2 Quad CPU Q9550 @ 2.83 GHz. MAMMOTH and MATT were run with their default parameter settings.

## Methods

In this section, we provide the mathematical and algorithmic framework underlying MAPSCI. As mentioned earlier MAPSCI shares common elements with the algorithm in [5], and therefore, we follow the same general outline. However, we only present the full details when there are significant differences and refer the reader to [5] when there is an overlap.

### Multiple Structure Alignment: Problem Formulation

Let {*P*_1_, *P*_2_, ⋯, *P*_*k*_} be the given set of *K *proteins and let *l*_*i *_be the number of *C*_*α *_atoms along the backbone of protein *P*_*i*_. We represent *P*_*i *_as a sequence of *coordinate triples *, 1 ≤ *j ≤ l*_*i*_, that represent the coordinates of the *j*th *C*_*α *_atom of *P*_*i *_along the backbone. (As is customary [14,15], we consider only the backbone, not the amino acid residues themselves.) Let *P*_0 _= , ⋯,  denote the *consensus structure*, of length *l*_0_.

A *correspondence *of the *K *proteins in  and the consensus structure *P*_0 _can be represented as a matrix *H *= ()_0 ≤ *i *≤ *K*,1 ≤ *j *≤ *L*_, for some *L *≥ max_0 ≤ *i *≤ *K*_{*l*_*i*_}, where  is either a coordinate triple belonging to the *i*th protein or a *gap*. Distances between coordinate triples are based on the squared distance between them in ℝ^3^. The distance between a coordinate triple and a gap is called a *gap penalty*, and is denoted by *ρ*.

The results reported in this paper use 16.0 for the value of the gap penalty.

Let *G*_*i *_= (*H*_*i *_- *T*_*i*_)*R*_*i *_= (*H*_*i *_- *e *× *t*_*i*_)*R*_*i*_, for *i >*0, where *R*_*i *_∈ ℝ^3 × 3 ^is some rotation matrix, *T*_*i *_= *e *×  is the translation matrix, *e *∈ ℝ^*L *× 1 ^is a vector with 1 in each entry, and  ∈ ℝ^1 × 3 ^is a translation vector. (The transformation of a gap remains a gap.) Note that *P*_0 _remains unchanged, i.e. *G*_0 _= *H*_0_.

Under the multiple structure alignment we define the *distance between the consensus structure P*_0 _*and protein P*_*j *_as , where *d*(·, ·) denotes the following distance function:

The distance between *P*_0 _and *P*_*j *_can be represented compactly as , where ||·||_*F *_denotes the *Frobenius norm *[16], with the additional convention that the squared difference between a coordinate triple and a gap is *ρ*^2^. The total distance of the *K *proteins to the consensus structure, called the *Sum-of-Consensus distance*, or *SC-distance*, is then defined as(1)

Intuitively, the *SC-distance *measures how well the consensus structure represents the given set of *K *proteins. A similar distance function is used in [17], where each protein is represented as a set of vectors in ℝ^4^.

We can now define the multiple structure alignment problem as follows:

### Multiple Structure Alignment Problem

*Given a set *{*P*_1_, *P*_2_, ⋯, *P*_*K*_} *of protein structures, compute a transformation (i.e., rotation and translation) for each protein, and generate a consensus structure P*_0_, *such that the resulting multiple structure alignment has minimum SC-distance as defined in Equation (1)*.

In the next section, we present a heuristic for this problem. Our algorithm approximates the global minimum of the *SC-distance *by iterative refinement of an initial multiple structure alignment and converges to a local minimum.

#### Step I: Choice of the initial consensus structure

We consider four choices for initial consensus structure: (i) *median protein*, i.e. the protein of median length; (ii) *center protein*, i.e. the protein that minimizes the sum of the pairwise distances to all the other proteins; (iii) the *minmax *protein, i.e. the protein with the smallest maximum pairwise distance; and (iv) *maxcore protein*, i.e. the protein that generates the largest initial core. (The first three choices for initial consensus are considered in [5].)

**Figure 5:**
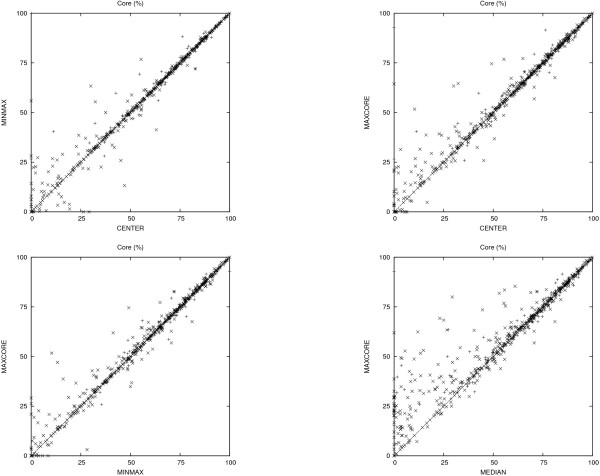
**Consensus choice comparison**. Comparison between the sizes of the aligned cores for different choices of initial consensus protein.

The experimental results in Figure 5 indicate that MAPSCI is quite robust in terms of the choice of initial consensus. However, the data suggests that the *median protein *occasionally leads to alignments with very low core size, and therefore is the least reliable choice. The other three choices seem to work well in practice, although they are more expensive computationally. The results reported in the **Comparison **section use the *maxcore protein *as the initial consensus.

#### Step II: Compute an initial correspondence

After we determine the consensus structure *P*_0 _in Step I, the *K *- 1 pairwise structure alignments between *P*_0 _and *P*_*i *_≠ *P*_0_, for *i *= 1, ⋯, *K*, are computed using the algorithm in [7]. (Other pairwise structure alignment algorithms could also be used instead.) The *K *- 1 pairwise structure are combined in Line 6 of the algorithm (Table 1) using the *center-star-like *method described in [5].

#### Step III: Compute optimal rotation and translation matrices and consensus structure

Given a correspondence *H *= () the objective is to find the rotation and translation matrices *R*_*j *_and *T*_*j*_, for *j *= 1, ⋯, *K*, and the consensus structure , such that the sum of the pairwise alignment distances between  and each (transformed) *P*_*j *_is minimum; i.e. we wish to minimize(2)

Direct minimization of *S *over , and the *T*_*j*_'s and *R*_*j*_'s seems difficult. Instead, we propose an iterative procedure for minimizing *S*. Within each iteration, the minimization of *S *is carried out in two stages that are interleaved: (1) computation of the optimal  for given *R*_*j*_'s and *T*_*j*_'s, and (2) computation of the optimal *R*_*j*_'s and *T*_*j*_'s for a given .

#### Computation of the optimal consensus structure

First, we show how to compute the consensus structure, given the rotation and translation matrices *R*_*j*_'s and *T*_*j*_'s, as stated in the following theorem:

**Theorem 1**. *Assume that the correspondence is represented as a matrix H *= () *and * = (*J*_1_, ⋯, *J*_*L*_)^*T *^*is the optimal consensus structure. For each column j, let I*_*n *_*be the set of indices of proteins with a non-gap in the jth column and I*_*g *_*be the set of indices of proteins with a gap in the jth column. Then *, *in the jth position of the optimal consensus structure equals either the coordinate triple *, *or a gap*.

*Proof*. For each *j*, we consider two distinct cases for *J*_*j*_: either it is a coordinate triple, *x*, or a gap. If *J*_*j *_is a gap, then the sum of the distances between  and each protein *P*_*j *_along the *j*th column is |*I*_*n*_|*ρ*^2^, where *ρ *is the gap penalty. If *J*_*j *_is a coordinate triple, *x*, then the sum of the distances between  and each protein *P*_*j *_along the *j*th column is , which is minimized, for . Therefore, if , then the optimal choice for  is the coordinate triple *x*_*j*_; otherwise, the optimal choice for  is a gap.

#### Computation of the optimal translation matrix

In this section, we show how to compute the optimal translation matrix *T*_*i*_, for each *i*, for a given consensus structure . From Eq. (2), it is clear that the optimal *T*_*i *_and *T*_*j*_, for *i *≠ *j *are independent of each other. Hence, in the following, we focus on the computation of *T*_*i*_, for a specific *i*. The translation matrix *T*_*i *_can be decomposed as *T*_*i *_= *e *× *t*_*i*_, where *t*_*i *_∈ *R*^1 × 3 ^is the translation vector.

As mentioned earlier, the transformation of a gap remains a gap. Hence the computation of the translation and rotation matrices is independent of the mismatches (i.e., where at least one of the two elements being compared is a gap). We can thus simplify the computation by removing all mismatches in the alignment between the consensus structure  and the *i*th protein *P*_*i*_.

Let *A *∈ ℝ^*n *× 3 ^and *B *∈ ℝ^*n *× 3 ^consist of the coordinate triples from the consensus structure and the *i*th protein, respectively, after removing the mismatches. (Here *n *is the number of matches between the consensus structure and the *i*th protein, i.e., comparison of two non-gaps). Without loss of generality, assume *e*^*T*^*A *= [0, 0, 0], i.e., the coordinate triples in the consensus protein are centered at the origin. The optimal translation vector is the one that matches the centroids of the coordinate triple vectors from *A *and *B *as stated in the following theorem:

**Theorem 2**. *Let A and B be defined as above. Assume that e*^*T*^*A *= [0, 0, 0]. *Then for any rotation matrix R*_*i*_, *the optimal translation vector t*_*i *_*for minimizing **is given by *.

More details can be found in [18].

#### Computation of the optimal rotation matrix

Next, consider the rotation matrix *R*_*i*_. We can assume that the coordinate triple vectors from both A and B are centered at the origin. It follows that

Hence the minimum of *S*_*i *_is obtained when trace (*A*^*T*^*BR*_*i*_) is maximized.

Let the Singular Value Decomposition (SVD) [16] of *A*^*T*^*B *be *U*Σ*V*^*T*^, where *U *and *V *are orthogonal and Σ is diagonal.

**Theorem 3**. *The optimal rotation matrix R*_*i *_*that minimizes S*_*i *_= ||*A *- *BR*_*i*_||^2 ^*is given by R*_*i *_= *UWV*^*T*^, *where W *= *diag*(1, 1, 1), *if det*(*UV*^*T*^) = 1, *and W *= *diag*(1, 1, -1), *if det*(*UV*^*T*^) = -1.

More details can be found in [18].

#### Convergence of the algorithm

In this section, we show that MAPSCI converges, by showing that the SC-distance is non-increasing from one iteration to the next.

Recall that from Eq. (1),

Line 4 in MAPSCI decreases the distance between the consensus structure and each of the *K *proteins, since the dynamic programming produces an alignment with minimum cost. By the property of the center-star-like method, Line 6 leaves unchanged the distance between the consensus structure and each of the *K *proteins. By Theorems 2 and 3, the transformations computed in Line 7 do not increase the distance between the consensus structure and the *j*th protein, for each *j*. It is clear that Line 8 does not change the pairwise distance, since the cost for aligning two gaps is zero. Finally, by Theorem 1, Line 9 does not increase the sum of the pairwise distances from the consensus structure to the other proteins. Hence, the SC-distance is non-increasing, and the algorithm converges.

#### Complexity analysis

Let *n *be the maximum length of the *K *proteins. Then the overall running time of the algorithm is *O*(*K*^2^*n*^2^). (If we choose the initial consensus structure as the protein of median length, the running time is *O*(*Kn*^2 ^+ *K*^2^*n*).) The run time analysis is similar to that of the algorithm in [5].

## Conclusions

We have presented an algorithm, called MAPSCI, to compute a multiple structure alignment for a set of proteins, together with their consensus structure. The algorithm represents the input proteins and the consensus as sequences of coordinate triples and computes an approximation to the optimal multiple structure alignment that minimizes the sum of the pairwise distances between the consensus and each input protein. Experimental results on a benchmark datasets derived from the HOMSTRAD and SABmark databases show that the algorithm compares favorably with existing algorithms for multiple structure alignment (MAMMOTH and MATT).

## Availability and requirements

• Project name: MAPSCI

• Project home page: http://www.geom-comp.umn.edu/mapsci

• Operating system(s): Platform-independent

• Programming language: C++

• License: Free BSD

## Authors' contributions

JY contributed to the design of the algorithm and experiments, and drafting of the manuscript. II contributed to the experiments and the implementation of the algorithm. RJ contributed to the refinement of the algorithm and drafting of the manuscript. All authors read and approved the final manuscript.
